# The impact of drought on vegetation conditions within the Damqu River Basin, Yangtze River Source Region, China

**DOI:** 10.1371/journal.pone.0202966

**Published:** 2018-08-24

**Authors:** Zhilong Zhao, Yili Zhang, Linshan Liu, Zengzeng Hu

**Affiliations:** 1 Key Laboratory of Land Surface Pattern and Simulation, Institute of Geographic Sciences and Natural Resources Research, Chinese Academy of Sciences, Beijing, China; 2 University of Chinese Academy of Sciences, Beijing, China; 3 CAS Centre for Excellence in Tibetan Plateau Earth Sciences, Beijing, China; 4 College of Urban Economics and Public Administration, Capital University of Economics and Business, Beijing, China; Texas A&M University, UNITED STATES

## Abstract

Drought and vegetation conditions within the Damqu River Basin, part of the Yangtze River Source Region (YRSR), are assessed here using the standardized precipitation index (SPI), the standardized precipitation evapotranspiration index (SPEI), the normalized difference vegetation index (NDVI), and the leaf area index (LAI). We utilized Sen’s method, least squares regression method, linear regression and Pearson’s correlation analysis to study variations in drought and vegetation indices and the drought effect on vegetation between 1988 and 2015. Results reveal that droughts occurred at a 25% frequency over this period; SPI and SPEI analyses show that 1994, 1999, 2005, and 2010 were change points and that the basin was characterized by varying drought and humidity trends. Subsequent to 2010, both SPI and SPEI decreased within the basin, while 1995, 2000, 2004, and 2010 were change points for NDVI and LAI while the watershed exhibited variable trends in vegetation reduction and increase. The NDVI-annual values of 63.36% regions and the LAI-summer values of 68.39% areas within the basin were decreased during 1988–2015 and 2000–2015, respectively. Subsequent to 2010, both NDVI and LAI decreased within the basin and significant positive correlations at inter-annual and inter-summer time scales were seen in both drought and vegetation indices; drought has exerted a lag effect on vegetation as shown by significant positive correlations between annual SPI/SPEI values and following year NDVI/LAI values.

## Introduction

High-latitude regions are amongst the most sensitive to climate change globally [1–3]. As the “third pole” of the Earth, the water tower of Asia, and the national ecological security shelter zone of China [4–8], the Qinghai-Tibet Plateau faces the dual threats of climate change and human development [8–9]. The Three River Source Region (TRSR) within the hinterland of the Qinghai-Tibet Plateau is one of the largest areas of natural wetland at highest altitudes nationally and is often referred to as the “Chinese Water Tower”. This region is also critical for national ecological security and water conservation even though it comprises a vulnerable and sensitive ecological environment. The Damqu River Basin is located in the southwest of the Three River Source Nature Reserve and is the southern source of the Yangtze River.

China experiences frequent and severe droughts [10–12] that are especially marked within the TRSR [13]. Climate data for the period between 1971 and 2010 reveals that the TRSR can nevertheless be characterized by significant regional differences; although the climate of northern and eastern regions has been predominantly warm and humid since the 1990s, southern and western areas have remained mainly warm and dry [14]. It is also the case that global warming scenarios predict that droughts are likely to become more frequent and severe in the future [15]; these events negatively impact the growth of grasslands, reducing their productivity [16], and are a major reason for land degradation [17–20]. Droughts also represent a considerable threat to the sustainable development of animal husbandry economic systems, especially on grasslands [16]. This is relevant because one previous field study carried out within the Damqu River Basin between 2015 and 2016 showed that southwestern region of the TRSR is susceptible to aridification [21]. The American Meteorological Society classified drought-related phenomena into four types, meteorological, agricultural, hydrological, and socioeconomical [22]. Researchers often apply a variety of indices in this context to characterize complex drought phenomena, the most common of which include the standardized precipitation index (SPI) [23], the standardized precipitation evapotranspiration index (SPEI) [10,16,24–28], the Palmer drought severity index (PDSI) [29,30], the integrated drought condition index (IDCI) [31], the standardized vegetation index (SVI) [32], the optimized meteorological drought index (OMDI), and the optimized vegetation drought index (OVDI) [33]. The first two of these, the SPI and SPEI, have the advantage of incorporating multiple time scales (i.e., one month, three months, six months, 12 months, 24 months, and 36 months [24]) and are therefore more suitable than their counterparts for assessing the sensitivity of vegetation communities to water deficits [34]. In addition, the SPEI also evaluates the positive influence of precipitation and the negative influence of evapotranspiration on drought intensity [16,24] while at the same time remaining sensitive to the negative influence of temperature in the same way as the SPI and PDSI [16].

Our current understanding of how ecosystems respond to droughts remains incomplete and requires further research [35]. Variations in vegetation dynamics are often used to characterize ecosystem changes and are one important proxy that can be used to gain an in-depth understanding of global system fluctuations, especially in the context of climate change [35]. We therefore used the normalized difference vegetation index (NDVI) and leaf area index (LAI) in this study to characterize vegetation conditions within the Damqu River Basin in addition to the SPI and the SPEI to characterize drought conditions. We explored the responses of vegetation dynamics to drought conditions within the Damqu River Basin on the basis of these indices, initially assessing the inter-annual variability of events over a timescale of 12 months. We also assessed the inter-summer variability of droughts over a three-month timescale encompassing the wettest season of the year.

## Materials and methods

### Study area

The Damqu River is 331 km long and has a basin perimeter enclosed within 92.14° - 94.62° E and 32.40° - 33.96° N. This river basin is located at elevations between 4,503 m and 5,947 m and encompasses a total area of about 1.67 × 10^4^ km^2^. This region has a continental climate, with an average annual temperature of -1.05°C and an annual precipitation of 444.53 mm; records from the China Meteorological Data Center (CMDC) collected between 1998 and 2015 show that 60% of total precipitation occurs in the summer, in June, July, and August. These observations also show that the rate of annual mean temperature increase has been 0.07°C per year (significance (α) = 0.001), and that values were enhanced significantly between 2003 and 2015. Similarly, the annual precipitation increase rate has been 2.32 mm per year (α = 0.05); 1994, 1999, 2005, and 2010 were times when trends changed, and precipitation tended to either decrease or increase between these points [21]. We have shown in previous work that the area of wetlands decreased within the Damqu River Basin between 1988 and 2015 [21], and assume that drought is the main reason for this reduction. In order to test this hypothesis, we used the same timescale (between 1988 and 2015) to assess drought and vegetation condition.

**Fig 1:**
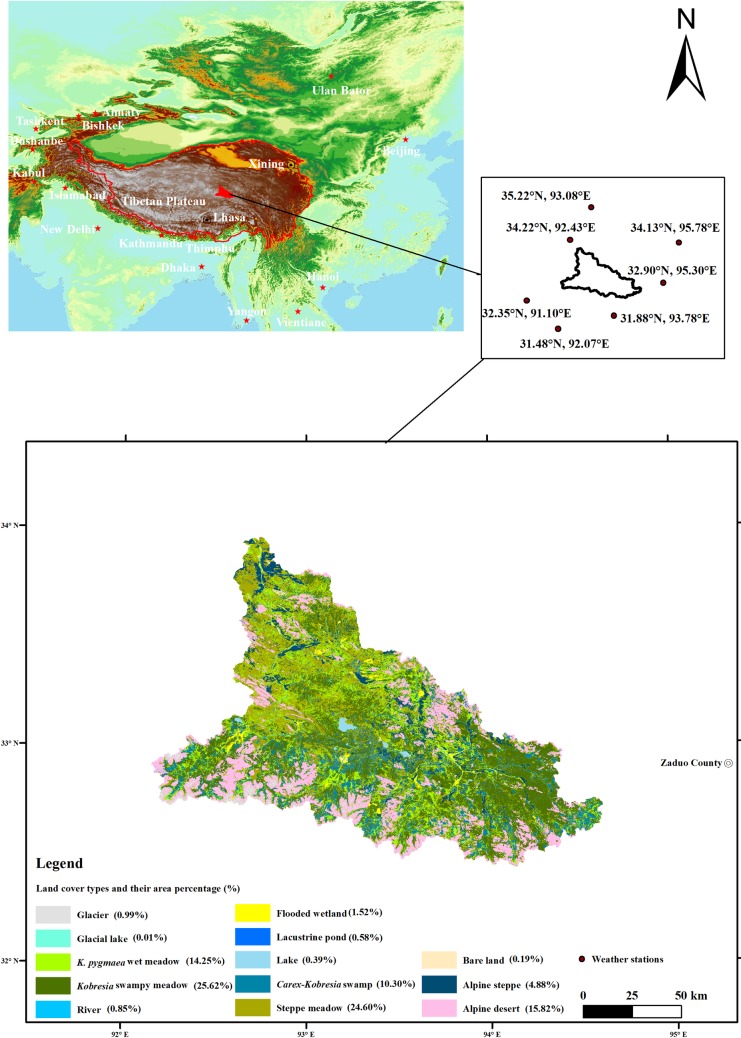
Map to show the location and land cover distribution of the Damqu River Basin watershed.

A total of 13 land-cover types can be defined within the Damqu River Basin including *Kobresia* swampy meadow, steppe meadow, alpine desert, *K*. *pygmaea* wet meadow, *Carex-Kobresia* swamp, alpine steppe, flooded wetland, glacier, river, lacustrine pond, lake, bare land, and glacial lake (Fig 1). Land cover data for this region in 2015 [21] show that the area of *Kobresia* swampy meadow accounted for the highest proportion (25.62%), while glacial lakes just cover 0.01% of the basin. The total area of the six main land cover types (i.e., *Kobresia* swampy meadow, steppe meadow, alpine desert, *K*. *pygmaea* wet meadow, *Carex-Kobresia* swamp, and alpine steppe) accounted for 95.47% of the basin area in 2015.

### Data sources

#### Remote sensing (RS) data

The RS data utilized in this study includes a Global Inventory Monitoring and Modeling Systems (GIMMS3g) NDVI dataset, a MOD13A2 NDVI dataset, and a MOD15A2 LAI dataset (Table 1). We used two of these datasets (GIMMS3g and MOD13A2) to compute NDVI changes and to assess whether, or not, they exhibit the same characteristics during the same period.

1. The GIMMS3g NDVI dataset used in this study was downloaded from https://ecocast.arc.nasa.gov/. Data were subjected to maximum value synthesis using the maximum value composite (MVC) approach in order to mitigate interference from clouds, the atmosphere, and solar altitude angles [36]. Annual NDVI was defined using average values for the 12 months in each calendar year.
2. Variation in NDVI across the growing season (i.e., June, July, and August, the wettest season of the year) between 2000 and 2014 was extracted from the MOD13A2 data product (https://ladsweb.nascom.nasa.gov/data/search.html). These data were also subjected to MVC analysis to minimize interference from clouds, the atmosphere, and solar altitude angles [37]. In this case, annual growing season NDVI was defined using average values for the period between May 25^th^ and August 28^th^ of each calendar year.
3. Variations in LAI throughout the growing season (i.e., June, July, and August) between 2000 and 2015 were calculated using MOD15A2 data (ftp://ftp.glcf.umd.edu/glcf/GLASS/LAI/MODIS/). Interference due to cloud pollution and the atmosphere were removed and missing data were supplemented [38]; annual growing season LAI was defined using average values for the period between May 25^th^ and August 28^th^ of each calendar year.

**Table 1:** The main characteristics of RS products used in this study.

Data	Data product	Spatial resolution	Temporal resolution	Time period
NDVI	GIMMS3g NDVI	8km	15 days	Between 1988 and 2015
NDVI	MOD13A2	1 km	16 days	May 25^th^ and August 28^th^ between 2000 and 2014
LAI	MOD15A2	1 km	8 days	May 25^th^ and August 28^th^ between 2000 and 2015

#### Meteorological data

The meteorological data used in this study were acquired from the CMDC (http://data.cma.cn/site/index.html) and include monthly mean air temperature and precipitation between 1988 and 2015. All data downloaded from the CMDC were evaluated using strict quality controls and checks, tested for erroneous entries, and subjected to homogeneity correction by the National Meteorological Information Center of China. We selected seven weather stations (S1 Table) in the vicinity of the Damqu River Basin for meteorological data collection as there are none within this region (Fig 1). Average monthly precipitation values were used to calculate the SPI, while average monthly temperature and precipitation values were used to calculate the SPEI.

### Methods

#### SPI and SPEI

The SPI was developed by Mckee et al. [39] and is one of the key indicators that is widely used to characterize variations in meteorological and hydrological droughts. We used the calculation software *spi_sl_6*.*exe* (http://drought.unl.edu/MonitoringTools/DownloadableSPIProgram.aspx) provided by the National Drought Mitigation Center to calculate the SPI.

The SPEI was developed by Vicente-Serrano et al. [24] and is also commonly used to characterize drought variation. This index was calculated using the software provided by Vicente–Serrano et al. [24] (http://digital.csic.es/handle/10261/10002).

#### Statistical analyses

We used the ratio of a region involved droughts (*P*_*i*_) to assess the frequency of drought [40,41]:

$$P_{i}=(n/N)\times 100\%$$ (1)

Where *n* is the number of the years in a region that involved droughts based on SPI or SPEI (SPI or SPEI ≤ −0.50) [16,27], *N* is the number of the years during 1988 to 2015, and *i* is the code for a region.

We used the nonparametric Mann-Kendall test for detecting the significance of the increasing or decreasing trend and the nonparametric Sen’s method for evaluating the slope of a linear trend. The Mann-Kendall test requires at least 4 values and calculation of the confidence intervals for the Sen’s slope estimate requires at least 10 values in a time series of values of meteorological elements [42,43].

We used the least squares method in order to detect trends in each index and to perform piecewise linear fitting of the SPI, the SPEI, the NDVI, and the LAI. This approach has been applied in previous work to identify overall trends and to compute the breakpoints between periods with significantly different tendencies [44]. We applied the statistical procedure and operation code outlined by Tomé and Miranda [44] in this study using the software ENVI/IDL to identify trend change points in drought and vegetation time series. These points divided the drought and vegetation time series in this study into some sub-periods, which can help find the stage characteristics in different sub-periods. We then performed a correlation analysis once these points had been obtained to assess the statistical significance of SPI, SPEI, NDVI, and LAI values.

We used the slope of as the index to detect the trend in vegetation dynamics for every pixel by using the least squares method, which can synthetically reflect the spatiotemporal change characteristics of vegetation coverage [45]. The slope is calculated as follows [45]:

$$Slope=\frac{n\times \sum_{i=1}^{n}i\times {(NDVI}_{i}or{LAI}_{i})-(\sum_{i=1}^{n}i)(\sum_{i=1}^{n}{(NDVI}_{i}or{LAI}_{i}))}{n\times \sum_{i=1}^{n}i^{2}-{\left(\sum_{i=1}^{n}i\right)}^{2}}$$ (2)

where *Slope* is the trend of vegetation coverage variation, *n* is the number of the years that RS products used in this study have crossed, *i* is the order of year from 1 to *n*, and *NDVI*_*i*_ or *LAI*_*i*_ is the value of NDVI or LAI in the *i*th year. When *Slope*>0, the NDVI or LAI shows an increasing trend, and when *Slope*<0, the NDVI or LAI shows a decreasing trend [45].

We calculated Pearson’s correlation coefficients between drought and vegetation indices at three and 12 month time scales and assessed their statistical significance at 0.01, 0.05, and 0.1 confidence levels for different months and years.

## Results

### SPI variation

**Fig 2:**
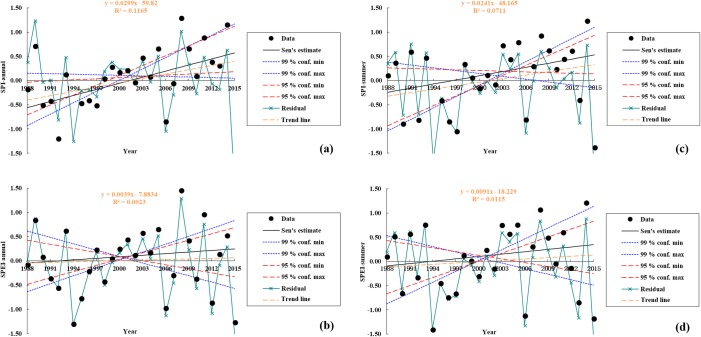
Variations in the SPI obtained from average monthly precipitation values and SPEI obtained from average monthly temperature and precipitation values within the Damqu River Basin between 1988 and 2015 (a) SPI-annual (b) SPEI-annual (c) SPI-summer (d) SPEI-summer.

Inter-annual variation in the SPI between 1988 and 2015 (Fig 2A) shows that droughts occurred in seven years according to the standard in Table 2 within the Damqu River Basin, at a 25% frequency. Data show that mild droughts occurred in 1990, 1995, 1997, and 2006, a moderate drought occurred in 1992, and severe droughts occurred in 1994 and 2015. Based on the Sen’s estimate, the values of SPI-annual showed a rise with a linear rate of 0.03/a during 1988–2015, and it passed the significance level α = 0.05 through Mann-Kendall test (Fig 2A).

**Table 2:** SPI and SPEI categories [16,27].

Categories	SPI/SPEI values
Extremely dry	Less than -2.00
Severely dry	Between -1.50 and -1.99
Moderately dry	Between -1.00 and -1.49
Light dry	Between -0.50 and -0.99
Near normal	Between 0.50 and -0.49
Humid	Greater than 0.50

**Fig 3:**
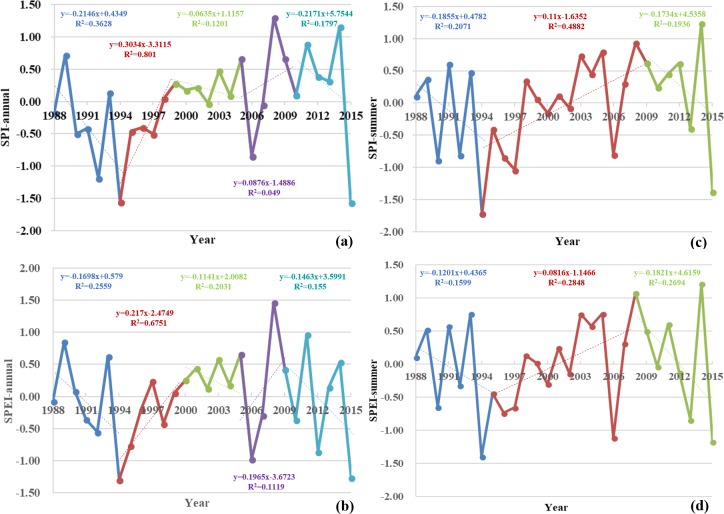
Point analysis of change trends and stage characteristics in the SPI and SPEI within the Damqu River Basin between 1988 and 2015 (a) SPI-annual (b) SPEI-annual (c) SPI-summer (d) SPEI-summer.

The least squares method was used to obtain trend change points in SPI time series, and then we detected the stage characteristics of the SPI between these points. Trend point analysis results show that changes of SPI-annual occurred in 1994, 1999, 2005, and 2010 (Fig 3A); SPI decreased between 1988 and 1994 (Pearson’s correlation coefficients (*r*) = -0.601, significance (*p*) = 0.077), indicating an increased drought trend during this period. In contrast, SPI increased between 1994 and 1999 (*r* = 0.896, *p* = 0.008), indicating an increased humidification trend during this period, while this index decreased again between 1999 and 2005 (*r* = -0.346, *p* = 0.201) indicating an enhanced drought trend. SPI also increased between 2005 and 2010 (*r* = 0.219, *p* = 0.338) indicating enhanced humidification during this period, while values of this index decreased from 2010 (*r* = -0.422, *p* = 0.202), indicating the more risk of drought.

Inter-summer (between June and August) variation in the SPI over the time period of this study (Fig 2C) shows that droughts occurred in seven years at a 25% frequency. Mild droughts occurred in 1990, 1992, 1996, and 2006, moderate droughts occurred in 1997 and 2005, and severe droughts occurred in 1994. Based on the Sen’s estimate, the values of SPI-summer showed an increase with a linear rate of 0.02/a during 1988–2015, and it passed the significance level α = 0.1 through Mann-Kendall test (Fig 2C).

Point analysis shows that trend changes of SPI-summer occurred in 1994 and 2009 (Fig 3C), while inter-summer SPI conformed to a downward trend between 1988 and 1994 (*r* = -0.447, *p* = 0.158), indicating the more risk of drought during this period. Inter-summer SPI increased between 1994 and 2009 (*r* = 0.698, *p* = 0.001) indicating a trend towards higher humidification, while inter-summer SPI decreased from 2009 onwards (*r* = -0.445, *p* = 0.158), indicating an increased risk of drought during this period.

### SPEI variation

Inter-annual variation in the SPEI between 1988 and 2015 (Fig 2B) shows that droughts occurred in six years according to the standard in Table 2 within the Damqu River Basin, at a 21.42% frequency. Data show that mild droughts occurred in 1992, 1995, 2006, and 2012, while moderate droughts occurred in 1994 and 2015. Based on the Sen’s estimate, the values of SPEI-annual showed a slight rise during 1988–2015, and it did not pass the significance level α = 0.1 through Mann-Kendall test (Fig 2B).

The least squares method was used to obtain trend change points in SPEI time series, and then we detected the stage characteristics of the SPEI between these points. Point analysis shows that trend changes of SPEI-annual occurred in 1994, 2000, 2005, and 2009 (Fig 3B); indeed, inter-annual SPEI followed a downward trend between 1988 and 1994 (*r* = -0.504, *p* = 0.124), indicating an enhanced risk of drought during this period, while an increase in inter-annual SPEI between 1994 and 2000 is indicative of an increased humidification trend (*r* = 0.821, *p* = 0.012). Inter-annual SPEI again decreased (*r* = -0.452, *p* = 0.154) between 2000 and 2005, indicating the more risk of drought, while inter-annual SPEI increased again (*r* = 0.336, *p* = 0.290) between 2005 and 2009, evidencing an enhanced humidification trend. Inter-annual values for this index then decreased from 2009 onwards (*r* = -0.396, *p* = 0.189), indicating the more risk of drought.

Inter-summer (between June and August) variation in the SPEI over the time period of this study (Fig 2D) reveals that droughts occurred in seven years within the basin, at a 25% frequency. Data show that mild droughts occurred in 1990, 1996, 1997, and 2013, while moderate droughts occurred in 1994, 2006, and 2015. Based on the Sen’s estimate, the values of SPEI-summer showed a slight increase during 1988–2015, and it did not pass the significance level α = 0.1 through Mann-Kendall test (Fig 2D).

Point analysis shows that trend changes of SPEI-summer occurred in 1995 and 2008 (Fig 3D); inter-summer SPEI values declined between 1988 to 1995 (*r* = -0.385, *p* = 0.173), indicating an enhanced drought trend throughout this period, while inter-summer SPEI values increased between 1995 and 2008 (*r* = 0.539, *p* = 0.023), indicating enhanced humidification. Inter-summer SPEI values decreased again from 2008 onwards (*r* = -0.534, *p* = 0.086), further evidencing the more risk of drought.

### NDVI variation

Variations in the NDVI are considered separately here on the basis of GIMMS and MODIS data.

#### NDVI based on GIMMS data

We explore NDVI variation within the Damqu River Basin between 1988 and 2015 from three perspectives in this section, changes in average values, variation slope of different values and proportional variations within each group with different values. Average values in this case mainly denote overall NDVI trends within the watershed throughout the study period; thus, annual NDVI data were uniformly graded to explore tendencies in variation within high-value and low-value regions in each year and to analyze differences in vegetation growth within the Damqu River Basin. Variation slope of annual NDVI is another aspect to detect spatial differences of vegetation index in the study area during 1988–2015.

**Fig 4:**
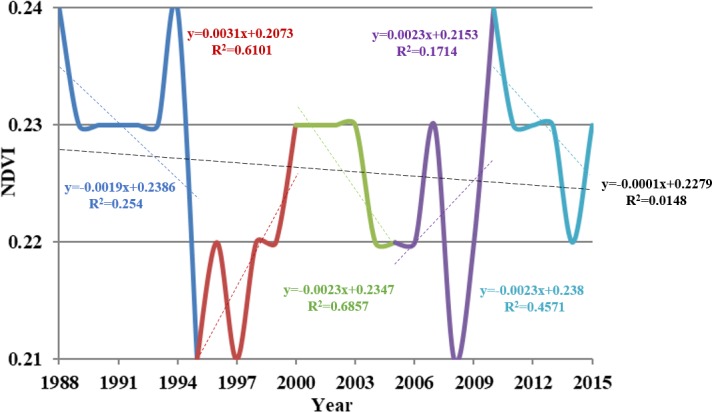
Variation in average annual NDVI values obtained from GIMMS3g and point analysis of trend changes within the Damqu River Basin between 1988 and 2015.

Data show that the average annual NDVI value was 0.23 and it showed a slight decrease (*r* = -0.122, *p* = 0.268) between 1988 and 2015 (Fig 4).

The least squares method was used to obtain trend change points in NDVI time series, and then we detected the stage characteristics of the NDVI between these points. The results of point analysis show that trend changes occurred in 1995, 2000, 2005, and 2010 (Fig 4); the average watershed value of NDVI decreased between 1988 and 1995 (*r* = -0.5040, *p* = 0.2090), indicating that vegetation was degraded over this period. In contrast, the average watershed NDVI value increased by 0.02 between 1995 and 2000 (*r* = 0.7811, *p* = 0.0666), but decreased again between 2000 to 2005 (*r* = -0.8281, *p* = 0.0418), demonstrating a trend towards degraded vegetation within the basin. Watershed NDVI increased again between 2005 and 2010 (*r* = 0.4140, *p* = 0.4144), but decreased from 2010 onwards (*r* = -0.6761, *p* = 0.1404), indicating further vegetation degradation.

**Fig 5:**
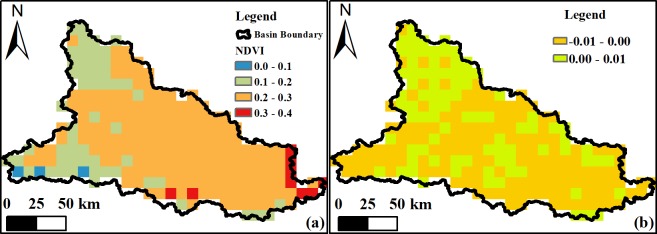
Average annual NDVI values (a) and NDVI-annual variation slope (b) within the Damqu River Basin between 1988 and 2015.

The condition of the Damqu River Basin as characterized by GIMMS data is reflected in a maximum NDVI value of 0.40 and a minimum value of 0.05 for the period between 1988 and 2015. A spatial map of average NDVI values for all regions within the basin is shown in Fig 5A, and another spatial map of variation slope of annual NDVI during 1988–2015 is exhibited in Fig 5B. In this period, the NDVI values of 63.36% regions within the basin was decreased, while the NDVI values of the 36.64% regions increased and these regions concentrated in the northwest and central part of the basin. Regional values on Fig 5A can be divided into four groups based on differences between minimum and maximum NDVI values. We then subsequently assessed whether, or not, the proportional changes seen in each group exhibit the same temporal variation characteristics compared to patterns in average NDVI value variation within the Damqu River Basin between 1988 and 2015.

**Fig 6:**
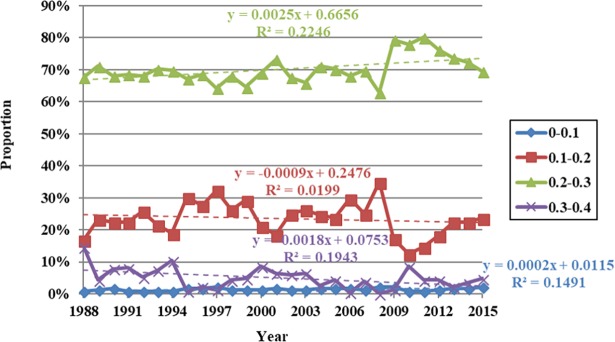
Proportional variation in annual NDVI values at different levels within the Damqu River Basin between 1988 and 2015.

The data presented in Fig 6 show that regional areas with NDVI values between 0.1 and 0.2 increased between 1988 and 1995 from 16.81% to 30.17%. This area then subsequently decreased between 1995 and 2000, a proportional decline of 21.12%, before increasing again between 2000 and 2005, a proportional expansion of 23.71%. This area then decreased to 12.50% between 2005 and 2010 before expanding again from 2010 onwards, reaching a proportion of 23.71% by 2015. Trends in regional areas with NDVI values between 0.2 and 0.3 exhibited the opposite behavior between 1988 and 2015 compared to those with values between 0.1 and 0.2, while those with values between 0.3 and 0.4 fluctuated and decreased over this period, an overall decrease in area proportion from 14.66% to 4.74%. Regions with NDVI values between 0.1 and 0.2 obviously expanded subsequent to 2010, while those with values between 0.2 and 0.3 followed a reversed trend. Proportional changes in regions with NDVI values between 0.3 and 0.4 exhibited the same temporal characteristics of variation as average values within the Damqu River Basin between 1988 and 2015. It is therefore clear that these changes influenced variation in average NDVI values across the whole basin.

#### NDVI based on MODIS data

We explore NDVI variation within the Damqu River Basin between 1988 and 2015 from the same three perspectives as discussed for GIMMS data, changes in average values, variation slope of different values and proportional variations within each group with different values. Average values are reported here in the same way as for GIMMS data.

**Fig 7:**
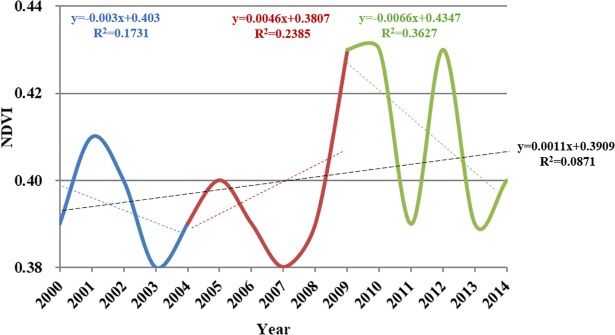
Variations in average summer NDVI values obtained from MOD13A2 and point analysis of trend changes within the Damqu River Basin between 2000 and 2014.

Data show that between 2000 and 2014, the average summer NDVI value was 0.40 and it showed a slight increase (*r* = 0.295, *p* = 0.143) (Fig 7).

Point analysis shows a change between 2004 and 2009 (Fig 7). Average watershed NDVI values between 2000 and 2004 tended to decrease (*r* = -0.4160, *p* = 0.4860), indicating vegetation degradation over this period, and this change characteristic is consistent with the annual NDVI value obtained from GIMMS3g in the same period. Average values increased between 2004 and 2009 (*r* = 0.4884, *p* = 0.3257) to 0.43, and this variation characteristic is consistent with the annual NDVI value between 2005 and 2009. Then the average values decrease again between 2009 and 2014 (*r* = -0.6023, *p* = 0.2058), further evidencing vegetation degradation within the basin and this change characteristic is consistent with the annual NDVI value during 2010–2014.

**Fig 8:**
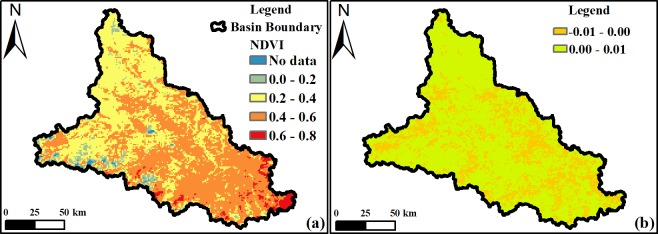
Average summer NDVI values (a) and NDVI-summer variation slope (b) within the Damqu River Basin between 2000 and 2014.

Summer NDVI values for the Damqu River Basin based on MODIS data reveal a maximum value of 0.77 and a minimum value of -0.18 for the period between 2000 and 2014. A spatial map of average summer NDVI values for all regions within the basin is presented in Fig 8A, and another spatial map of change slope of summer NDVI during 2000–2014 is showed in Fig 8B. In this period, the NDVI values of 25.46% regions within the basin was decreased, while the NDVI values of the 74.54% regions increased. Then we divided the values for all regions into five groups (Fig 8A) based on differences between minimum and maximum summer NDVI values and assessed whether, or not, proportional changes within each exhibit the same temporal variations compared to fluctuations in average records between 2000 and 2014. It is also noteworthy that the NDVI categories shown in Fig 8A differ from those in Fig 5A because of the use of different data products; these two figures can therefore be used to analyze regional NDVI differences within the Damqu River Basin.

**Fig 9:**
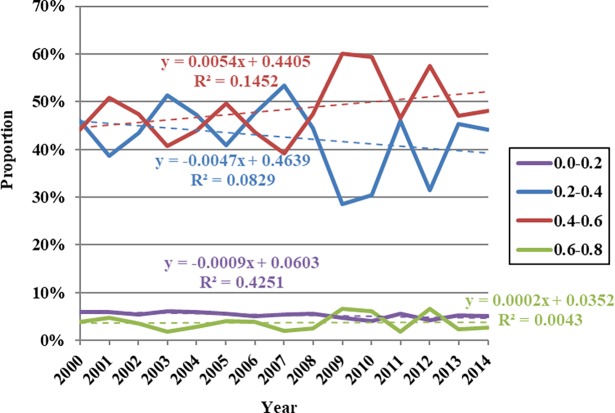
Proportional variation in summer NDVI values at different levels within the Damqu River Basin between 2000 and 2014.

The data presented in Fig 9 show that the regional area characterized by summer NDVI values between 0.2 and 0.4 increased between 2000 and 2004, from 46.08% to 47.27%. Subsequently, this area decreased between 2004 and 2009 to 28.56%, and then increased again from 2009 onwards, reaching 44.07% in 2014. In contrast, summer NDVI values between 0.4 and 0.6 between 2000 and 2014 followed the opposite trend, while regional area with values between 0.6 and 0.8 fluctuated to some extent. This area fluctuated and increased between 2000 and 2009 as the proportion of such regions within the watershed area increased from 3.97% to 6.62%. The proportion of this area decreased from 2009 onwards to 2.72% by 2014; data show that regions with NDVI values between 0.2 and 0.4 have expanded over time, while those with values between 0.4 and 0.6 have followed the opposite trend. Proportional changes in regions with NDVI values between 0.4 and 0.6 have exhibited the same temporal variations as average values within the Damqu River Basin between 2000 and 2014; it is clear that these changes have influenced variations in average NDVI values across the whole basin.

### Variation in the LAI

Trends in the LAI within the Damqu River Basin between 2000 and 2015 are explored here from three perspectives, changes in average values, variation slope of different values and proportional variations within each group with different values. Average values in this case mainly denote overall trends within the watershed over the study period, while LAI data were uniformly graded in order to explore variations in both high- and low-value regions in each year and to analyze differences in vegetation growth and intensity within the Damqu River Basin. Variation slope of LAI is another aspect to detect spatial differences of vegetation index in the study area during 2000–2015.

**Fig 10:**
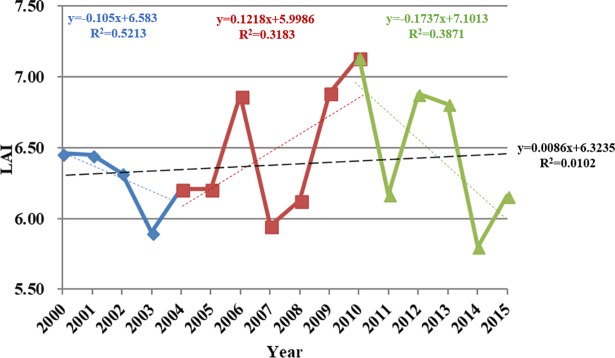
Variation in average summer LAI values obtained from MOD15A2 and point analysis of trend changes within the Damqu River Basin between 2000 and 2015.

Data show that the average summer LAI value was 6.40 and it showed a slight increase (*r* = 0.101, *p* = 0.355) between 2000 and 2015 (Fig 10).

The least squares method was used to obtain trend change points in LAI time series, and then we detected the stage characteristics of the LAI between these points. Point analysis reveals that trend changes occurred in 2004 and 2010 (Fig 10). Average watershed LAI values between 2000 and 2004 tended to decrease (*r* = -0.7220, *p* = 0.1684), indicating vegetation degradation over this period. Average watershed LAI values increased, however, between 2004 and 2010 (*r* = 0.5642, *p* = 0.1871) to 0.93, but decreased again between 2010 and 2015 (*r* = -0.6221, *p* = 0.1872); this latter trend is indicative of degraded vegetation within the basin.

**Fig 11:**
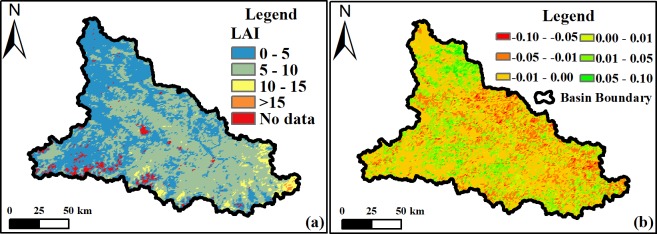
Average summer LAI values (a) and LAI-summer variation slope (b) within the Damqu River Basin between 2000 and 2015.

Damqu River Basin MODIS data results reveal a maximum summer LAI value of 27 and a minimum value of zero between 2000 and 2015. A spatial map of average summer values for all regions across the basin is presented in Fig 11A, and another spatial map of variation slope of summer LAI during 2000–2015 is showed in Fig 11B. In this period, the LAI values of 68.39% regions within the basin was decreased, while the LAI values of the 31.61% regions increased and these regions concentrated in the northwest and south part of the basin. These data on Fig 11A show that the proportional area of regions with LAI values greater than 15 accounts for 0.15% of the whole basin. We therefore divided all LAI values for all regions into five groups (Fig 11A), based on differences between minimum and maximum summer values and their proportional areas. We then assessed whether, or not, proportional changes for each group exhibit the same temporal characteristics of variation when compared to average summer values across the Damqu River Basin between 2000 and 2015.

**Fig 12:**
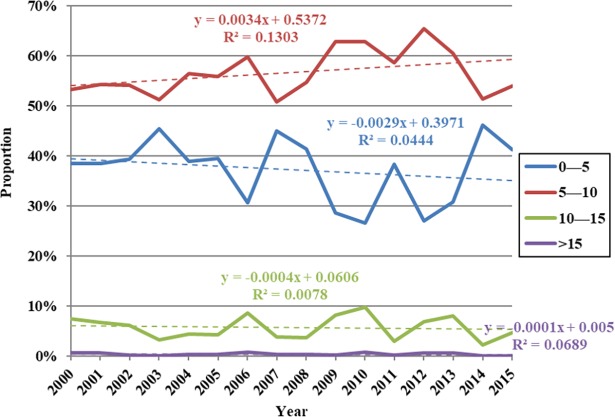
Proportional variation in summer LAI values at different levels within the Damqu River Basin between 2000 and 2015.

The data presented in Fig 12 show that the regional area characterized by summer LAI values up to five increased between 2000 and 2004, but decreased between 2004 and 2010; this area proportion decreased overall from 38.89% to 26.61%. This area then increased again after 2010, reaching 41.27% by 2015. In contrast, trends in the proportion of LAI values between five and ten between 2000 and 2015 followed the opposite pattern compared to their counterparts up to five, while the area characterized by values more than ten fluctuated slightly. The proportional area of these regions within the watershed then decreased from 8.15% to 4.70% between 2000 and 2015; from 2010 onwards, regions characterized by LAI values up to five tended to expand, while those characterized by values between five and ten expanded relatively obviously and those between five and ten contracted. Proportion changes in regional areas characterized by LAI values less than five have exhibited the same temporal characteristics as average values within the Damqu River Basin between 2000 and 2015; it is clear that these changes have influenced variations in average LAI values across the whole basin.

### Correlation between SPI and NDVI/LAI

Observations show that vegetation coverage within the Damqu River Basin increased in humid years and decreased in arid ones. Data show that 2009 vegetation correspond with peak drought indices (Fig 9), while the trough seen in 1995 corresponds to the same pattern in drought indices (Fig 6).

We performed a correlation analysis on SPI values for each year versus NDVI and LAI values for the same year. Results, however, revealed no significance at *p* = 0.1; the same result was also recovered for SPI values for each year versus LAI values for the same year. At the same time, analyses show that SPI values are negatively correlated with both NDVI and LAI values; a higher and more positive SPI value equates to more humid meteorological conditions that are increasingly beneficial for vegetation growth, while no negative correlations between SPI and NDVI/LAI were recovered. We also performed a correlation analysis to evaluate SPI values for each year versus NDVI/LAI values for the following year (Table 3).

**Table 3:** Pearson’s correlation coefficients for vegetation indices and SPI values within the Damqu River Basin.

Vegetation index	Vegetation index dataset	Period	SPI zero year lag	SPI one year lag	SPI two year lag
NDVI-annual	GIMMS3g	1988–2015	-0.247 (*p* = 0.103)	0.257*	0.324*
NDVI-summer	MOD13A2	2000–2014	0.069 (*p* = 0.404)	0.734***	0.129 (*p* = 0.338)
LAI-summer	MOD15A2	2000–2015	-0.205 (*p* = 0.223)	0.593***	0.244 (*p* = 0.201)

Notes

* α = 0.1 significance

*** α = 0.01 significance.

The results presented in Table 3 reveal significant positive correlations between SPI and NDVI/LAI over a one year lag temporal scale. At the same time, however, responses in vegetation indices (NDVI/LAI) to SPI exhibited time lag phenomena; both the NDVI and LAI exhibited significant positive responses to the SPI during summer, demonstrating that droughts have a more pronounced impact on values of these indices during the summer than on annual NDVI. The fact that the correlation between NDVI and SPI was significantly higher than between LAI and SPI shows that the former is more sensitive to variations in drought indices.

### Correlation between SPEI and NDVI/LAI

We performed additional correlation analyses to assess SPEI values in each year versus NDVI and LAI values for the same year. Results show that SPEI values for each year and NDVI values for the same year were not significantly correlated (*p* = 0.1); this was also the case for annual SPEI values and LAI values for the same year although values of the former were negatively correlated with both the NDVI and LAI. In this case, a higher and more positive SPEI value is indicative of more humid meteorological conditions, favoring plant growth. Data also reveal an absence of negative correlations between SPEI and NDVI/LAI; we therefore performed a correlation analysis to compare the SPEI values for each year versus NDVI/LAI values for the next (Table 4).

**Table 4:** Pearson’s correlation coefficients for vegetation indices and SPEI within the Damqu River Basin.

Vegetation index	Vegetation index dataset	Period	SPEI zero year lag	SPEI one year lag	SPEI two year lag
NDVI-annual	GIMMS3g	1988–2015	-0.248 (*p* = 0.102)	0.243 (*p* = 0.111)	0.391**
NDVI-summer	MOD13A2	2000–2014	-0.291 (*p* = 0.146)	0.693***	-0.034 (*p* = 0.456)
LAI-summer	MOD15A2	2000–2015	-0.404*	0.427**	0.333 (*p* = 0.122)

Notes

* α = 0.1 significance

** α = 0.05 significance

*** α = 0.01 significance.

Significant positive correlations exist between SPEI and NDVI/LAI at one year lag temporal scales (Table 4). At the same time, responses in vegetation indices (NDVI/LAI) to SPEI also exhibit time lag phenomena; both NDVI and LAI exhibited significant positive responses to the SPEI during the summer which shows that drought exerts a more pronounced impact on summer NDVI and LAI values than annual values of the former. Data show a significantly higher level of correlation between NDVI and SPEI than between LAI and SPEI; this suggests that NDVI is more sensitive to variations in drought indices.

## Discussion

Droughts are amongst most damaging of natural disasters from socioeconomic, environmental, and human perspectives [46]. Data show that the spatial extent of droughts in northern and eastern Asia increased between 1950 and 2000 [46], and that a dipole-type configuration with northerly droughts and southerly flooding was seen in eastern Asia between 1901 and 2014 [47]. The frequency of droughts has increased over the 21^st^ century [36] in most parts of China, while significant dry trends have been mainly seen in the southwest, north, northwest, and central parts of the country. The frequency of severe droughts in China remained extremely high in the 1990s and 2000s and drought frequency had increased in Tibetan Plateau since 1980s [10], while northern and eastern parts of the TRSR have experienced increased humidification since the 1990s and from the 21^st^ century onwards, respectively. In contrast, other parts of TRSR have experienced trends towards continuous droughts since the early 1980s; this has especially been the case in southern and western regions where wetness index linear trend rates have reached -0.8% per year [14]. As discussed, the Damqu River Basin is located within the southwestern TRSR; because of alternating dry-wet conditions throughout growing seasons between 2001 and 2010, most areas of the TRSR have been subject to humidification while the southern part of Zaduo County (containing the Damqu River Basin) has experienced a tendency towards droughts [48]. In this study, we also found the Damqu River Basin have experienced drought for six or seven times since the 1990s and from the 21^st^ century onwards. One consequence of drought is that the water status of this region has changed, with possible consequences for grassland productivity; declining grassland productivity constitutes a threat to the sustainable development of animal husbandry, the major economic activity within the Damqu River Basin. This watershed is also the southern source of the Yangtze River within China and comprises vast wetland areas; subsequent reductions in water contents have also negatively impacted the Damqu River Bain environment.

Vegetation cover in China has increased since the 1980s [49–51]. And Tibetan Plateau is also greening under the positive impact of climate change [51]. Data show that Tibetan Plateau NDVI values gradually increased between 1982 and 2012 up until the end of the 1990s and then decreased slightly in subsequent years [52] as a result of grassland variations [53]. The maximum NDVI recorded on this plateau increased in the summer (between June and August) by 0.4% over the ten years between 1982 and 2013 [54], while the spatial distribution of this index exhibits remarkable longitude zonality and increases continuously from west to east on the Tibetan Plateau [55]. Areas of grassland and forests decreased between 1990 and 2010 in the central high Himalayas [56]. Similarly, the area of degraded meadows in southern Qinghai Province increased 20.36% during 1976–2015 [57]. After the implementation of ecological protection measures [58,59] and the construction of the TRSR, more than 60% of the grassland area showed a significant improvement [60]. In light of the impacts of regional climate variation, vegetation dynamics also experience significant changes at the regional level; western and southern central regions have experienced vegetation degradation, for example, while northern and northwestern areas have seen increases [61]. Regions characterized by degraded vegetation are mainly distributed in Qumarleb and Zaduo counties within the eastern and southern parts of the TRSR [62–64], including the Damqu River Basin. In this study, we also found the NDVI-annual values of 63.36% regions within the Damqu River Basin was decreased during 1988–2015. In China, the growing-season (April–October) LAI (LAI_GS_) showed an increase (average trend of 0.0070/a, ranging from 0.0035/a to 0.0127/a) during 1982–2009 [49]. The average summer LAI value also showed a slight increase with a linear rate of 0.0086/a between 2000 and 2015 within the Damqu River Basin, even though it did not pass the significance level α = 0.1. Based on the satellite datasets of GIMMS, GLOBMAP and GLASS, the change trend of LAI_GS_ in most regions of the Damqu River Basin was between -0.01 and 0.01 during the period 1982–2009 [49]. In this study, we also found the variation slope of LAI-summer values of 61.03% regions within the Damqu River Basin was between -0.01 and 0.01 during 2000–2015 (Fig 11B).

We utilized two different datasets (i.e., GIMMS3g and MOD13A2) in this analysis to compute NDVI changes and generated different results [65]. Data show that while NDVI values for the Damqu River Basin fluctuated between 0.21 and 0.24 (GIMMS3g), watershed values ranged between 0.38 and 0.43 (MOD13A2). Changes in NDVI nevertheless exhibited the same temporal characteristics between 2000 and 2015 irrespective of dataset.

The results of this study corroborate previous related research in the identification of lag effects in vegetation responses to drought [16,36]. It is notable that the drought indices used in this study were calculated from precipitation and temperature data; it remains an open question whether, or not, lag effects within the Damqu River Basin are the result of temperature or precipitation and further research in this area will be required. Limitations in both the density and spatial distribution of meteorological data also raise uncertainties about the accuracy of SPI and SPEI results for the Damqu River Basin.

## Conclusions

We explored the dynamics of drought and vegetation cover across the Damqu River Basin in this study and assessed the impacts of the former on the latter between 1998 and 2015.

The results of this study show that both SPI and SPEI can be used as reliable indicators to characterize hydrological drought conditions within the Damqu River Basin. These two indicators exhibit the same temporal characteristics over the study period; between 1988 and 2015, droughts occurred at a frequency of 25%. Trend change points were seen in 1994, 1999, 2005, and 2010; indicators between these points exhibited both downward and upward trends when the watershed was subject to a higher frequency of droughts or increased humidification, respectively. Subsequent to 2010, both parameters decreased, indicating the more risk of drought within this basin.

Results show that both the NDVI and LAI can be used as indicators to characterize vegetation conditions within the Damqu River Basin; both these indices displayed the same temporal characteristics over the period of this study. The NDVI-annual values of 63.36% regions within the basin was decreased during 1988–2015. Similarly, the LAI-summer values of 68.39% regions within the basin was decreased between 2000 and 2015. Change points in trends were seen in 1995, 2000, 2004, and 2010; indicators at these times either decreased or increased when the watershed was subject to vegetation degradation or enhanced growth, respectively. The fact that both parameters decreased after 2010 is also indicative of degraded vegetation.

The data presented in this study reveal that vegetation coverage within the Damqu River Basin grew better in humid years while the opposite was the case in arid years. Drought and vegetation indices are both characterized by significant positive correlations at different time scales across the Damqu River Basin. It is noteworthy that a time lag effect in the responses of both NDVI and LAI vegetation indices was seen with respect to the SPI and SPEI drought indices.

## Supporting information

S1 Table Locations of weather stations in the vicinity of the Damqu River Basin. (DOCX)

S2 Table Detailed values of drought indices within the Damqu River Basin during 1988–2015. (DOCX)

S3 Table Detailed values of vegetation indices within the Damqu River Basin during 1988–2015. (DOCX)
