# Supplementary material for: The impact of drought on vegetation conditions within the Damqu River Basin, Yangtze River Source Region, China
Source: PLoS One. 2018 Aug 24;13(8):e0202966. doi: 10.1371/journal.pone.0202966 (PMC6108485; doi:10.1371/journal.pone.0202966)
Supplement: S1 Table — (DOCX) [file pone.0202966.s001.docx]

**S1 Table. Locations of weather stations in the vicinity of the Damqu River Basin.**

| ID of the weather station | Name | Longitude (°) | Latitude (°) | Altitude (m) |
| --- | --- | --- | --- | --- |
| 52908 | Wudaoliang | 93.08 | 35.22 | 4617 |
| 56018 | Zaduo | 95.30 | 32.90 | 4174 |
| 55294 | Anduo | 91.10 | 32.35 | 4696 |
| 56106 | Suoxian | 93.78 | 31.88 | 4005 |
| 55299 | Naqu | 92.07 | 31.48 | 4513 |
| 56004 | Tuotuohe | 92.43 | 34.22 | 4535 |
| 56021 | Qumalai | 95.78 | 34.13 | 4195 |
