# Supplementary material for: The impact of drought on vegetation conditions within the Damqu River Basin, Yangtze River Source Region, China
Source: PLoS One. 2018 Aug 24;13(8):e0202966. doi: 10.1371/journal.pone.0202966 (PMC6108485; doi:10.1371/journal.pone.0202966)
Supplement: S2 Table — (DOCX) [file pone.0202966.s002.docx]

**S2 Table. Detailed values of drought indices within the Damqu River Basin during 1988-2015.**

| Year | SPI-annual | SPEI-annual | SPI-summer | SPEI-summer |
| --- | --- | --- | --- | --- |
| 1988 | -0.17 | -0.07 | 0.1 | 0.1 |
| 1989 | 0.72 | 0.85 | 0.4 | 0.5 |
| 1990 | -0.50 | 0.09 | -0.9 | -0.6 |
| 1991 | -0.41 | -0.35 | 0.6 | 0.6 |
| 1992 | -1.19 | -0.55 | -0.8 | -0.3 |
| 1993 | 0.14 | 0.63 | 0.5 | 0.8 |
| 1994 | -1.55 | -1.29 | -1.7 | -1.4 |
| 1995 | -0.46 | -0.77 | -0.4 | -0.4 |
| 1996 | -0.40 | -0.20 | -0.8 | -0.7 |
| 1997 | -0.50 | 0.24 | -1.0 | -0.7 |
| 1998 | 0.05 | -0.42 | 0.3 | 0.1 |
| 1999 | 0.29 | 0.05 | 0.1 | 0.0 |
| 2000 | 0.18 | 0.26 | -0.2 | -0.3 |
| 2001 | 0.22 | 0.44 | 0.1 | 0.2 |
| 2002 | -0.03 | 0.13 | -0.1 | -0.1 |
| 2003 | 0.48 | 0.58 | 0.7 | 0.8 |
| 2004 | 0.09 | 0.18 | 0.4 | 0.6 |
| 2005 | 0.67 | 0.66 | 0.8 | 0.8 |
| 2006 | -0.84 | -0.97 | -0.8 | -1.1 |
| 2007 | -0.05 | -0.29 | 0.3 | 0.3 |
| 2008 | 1.30 | 1.46 | 0.9 | 1.1 |
| 2009 | 0.67 | 0.43 | 0.6 | 0.5 |
| 2010 | 0.10 | -0.36 | 0.2 | 0.0 |
| 2011 | 0.90 | 0.97 | 0.5 | 0.6 |
| 2012 | 0.39 | -0.86 | 0.6 | -0.1 |
| 2013 | 0.32 | 0.14 | -0.4 | -0.8 |
| 2014 | 1.17 | 0.53 | 1.2 | 1.2 |
| 2015 | -1.56 | -1.26 | -1.4 | -1.2 |
