# Supplementary material for: The impact of drought on vegetation conditions within the Damqu River Basin, Yangtze River Source Region, China
Source: PLoS One. 2018 Aug 24;13(8):e0202966. doi: 10.1371/journal.pone.0202966 (PMC6108485; doi:10.1371/journal.pone.0202966)
Supplement: S3 Table — (DOCX) [file pone.0202966.s003.docx]

**S3 Table. Detailed values of vegetation indices within the Damqu River Basin during 1988-2015.**

| Year | NDVI-annual | NDVI-summer | LAI-summer |
| --- | --- | --- | --- |
| 1988 | 0.24 |  |  |
| 1989 | 0.23 |  |  |
| 1990 | 0.23 |  |  |
| 1991 | 0.23 |  |  |
| 1992 | 0.23 |  |  |
| 1993 | 0.23 |  |  |
| 1994 | 0.24 |  |  |
| 1995 | 0.21 |  |  |
| 1996 | 0.22 |  |  |
| 1997 | 0.21 |  |  |
| 1998 | 0.22 |  |  |
| 1999 | 0.22 |  |  |
| 2000 | 0.23 | 0.39 | 6.46 |
| 2001 | 0.23 | 0.41 | 6.45 |
| 2002 | 0.23 | 0.40 | 6.32 |
| 2003 | 0.23 | 0.38 | 5.90 |
| 2004 | 0.22 | 0.39 | 6.21 |
| 2005 | 0.22 | 0.40 | 6.21 |
| 2006 | 0.22 | 0.39 | 6.87 |
| 2007 | 0.23 | 0.38 | 5.95 |
| 2008 | 0.21 | 0.39 | 6.13 |
| 2009 | 0.22 | 0.43 | 6.89 |
| 2010 | 0.24 | 0.43 | 7.14 |
| 2011 | 0.23 | 0.39 | 6.17 |
| 2012 | 0.23 | 0.43 | 6.88 |
| 2013 | 0.23 | 0.39 | 6.81 |
| 2014 | 0.22 | 0.40 | 5.80 |
| 2015 | 0.23 |  | 6.16 |
